# The Cell Cycle Checkpoint Regulator ATR Is Required for Internal Aluminum Toxicity-Mediated Root Growth Inhibition in *Arabidopsis*

**DOI:** 10.3389/fpls.2018.00118

**Published:** 2018-02-14

**Authors:** Yang Zhang, Jinliang Guo, Mo Chen, Lun Li, Lihua Wang, Chao-Feng Huang

**Affiliations:** ^1^College of Resources and Environmental Sciences, Nanjing Agricultural University, Nanjing, China; ^2^Shanghai Center for Plant Stress Biology, National Key Laboratory of Plant Molecular Genetics, CAS Center for Excellence in Molecular Plant Sciences, Chinese Academy of Sciences, Shanghai, China; ^3^Flower Research Institute, Yunnan Academy of Agricultural Sciences, Kunming, China

**Keywords:** aluminum toxicity, *Arabidopsis thaliana*, ATR, cell cycle checkpoint, DNA damage, external, internal

## Abstract

Aluminum (Al) can target multiple sites of root cells for toxicity, including the cell wall, the plasma membrane and symplastic components. Previous work revealed that the cell cycle checkpoint regulator (ATR) Ataxia Telangiectasia-mutated and Rad3-related is required for Al toxicity-induced root growth inhibition in *als3* and that the symplastic component DNA is an important target site of Al for the toxicity. However, whether monitoring DNA integrity through ATR-regulated pathway is required for Al-induced root growth inhibition in other Al-sensitive mutants remains unknown. In this study, we demonstrated that the *atr* mutation could also rescue the Al hypersensitivity and Al-induced cell cycle arrest in *star1*, which supports the hypothesis that ALS3 and STAR1 function together to be involved in the detoxification of Al in *Arabidopsis*. However, mutation of *ATR* could not rescue the Al-sensitive phenotype of *almt1* or *stop1*, both of which are defective in external detoxification mechanisms of Al. We further showed that the Al hypersensitivity and Al-induced quiescent center (QC) differentiation in *als1* could also be rescued by the *atr* mutation. Therefore, our results suggest that ATR-regulated pathway is involved in the modulation of internal Al toxicity-mediated root growth inhibition in *Arabidopsis*.

## Introduction

Aluminum (Al) comprises about 7% of the earth’s crust and is the most abundant metallic element. In neutral or alkaline soils, Al exists as insoluble aluminosilicates or oxides, which are non-toxic to plants. However, in acid soils with a pH of 5.5 or lower, solubilization of Al is enhanced and phytotoxic forms of Al are released into soil to levels that affect root growth. As a consequence, Al toxicity on acid soils becomes one of the most severe global problems since these soils comprise approximately 50% of the world’s potentially arable land (von Uexkull and Mutert, 1995; Kochian et al., 2004).

In acidic soils, Al exists as the octahedral hexahydrate Al(H_2_O)_6_^3+^, which is more commonly referred to as Al^3+^. The phytotoxic Al^3+^ is the hardest Lewis acid, which is characterized by a low covalent and a high ionic index. Hard metal ions have strong interactions with organic molecules bearing oxygen groups (Poschenrieder et al., 2008). Therefore, Al^3+^ preferentially binds to phosphate, sulfate, and carboxyl groups for toxicity. Considering the components of a plant cell, Al is believed to target multiple sites for toxicity, including the cell wall, the plasma membrane and inside the cells. Cell walls and intercellular spaces are the first sites of the root in contact with Al when the roots are exposed to Al. Many studies have shown that most of the Al is bound to the cell wall. The ratio of cell wall Al to the total Al has been reported to range from 85 to 99.9% (Ma, 2007). Al can also bind to the plasma membrane and alter the membrane fluidity and surface potential (Kinraide, 2001), block ion channel activity (Pineros and Kochian, 2001), and induce the reactive oxygen species (ROS) as well as lipid peroxidation on the plasma membrane (Yamamoto et al., 2001). Furthermore, a small portion of Al can enter the symplasm rapidly and may interact with a number of symplastic targets (Lazof et al., 1996; Silva et al., 2000). For example, Al disrupts the cytoskeleton by interacting with both microtubules and actin filaments (Grabski and Schindler, 1995; Blancaflor et al., 1998), and blocks signal transduction pathways, particularly in Ca^2+^ homeostasis and signaling (Jones and Kochian, 1995; Jones et al., 1998; Zhang and Rengel, 1999). Al can also interact with DNA (Karlik et al., 1980; Karlik and Eichhorn, 1989), which is expected to have serious effects on gene expression and chromosome structure.

To cope with Al toxicity, plants have evolved Al-resistance mechanisms, including external and internal detoxification of Al (Ma et al., 2001; Kochian et al., 2004). In *Arabidopsis thaliana*, external detoxification of Al is primarily achieved through AtALMT1-mediated secretion of malate to form a non-toxic form of Al-malate in the apoplast (Hoekenga et al., 2006), and the citrate transport AtMATE play a minor role in the external detoxification of Al (Liu et al., 2009). STOP1, a C2H2 transcription factor, is involved in the detoxification of Al mainly through the regulation of *AtALMT1* expression (Iuchi et al., 2007). For the internal detoxification of Al, the tonoplast-localized ATP-binding cassette (ABC) transporter ALS1 is required, which tolerates Al presumably via the transport of cytosolic Al into vacuoles (Larsen et al., 2007). STAR1 and STAR2/ALS3 encode a nucleotide-binding domain and transmembrane domain of a bacterial-type ABC transporter, respectively, and are suggested to be involved in Al tolerance through modification of cell wall or redistribution of Al from Al-sensitive root tips to other less Al-sensitive tissues (Larsen et al., 2005; Huang et al., 2009, 2010). Recently, Dong et al. (2017) reported that unlike rice STAR1 and STAR2, *Arabidopsis* ALS3 interacts with AtASTAR1 to be localized to the tonoplast, suggesting that AtSTAR1/ALS3 might be also required for the internal detoxification of Al.

Through the screening of the suppressors of the Al hypersensitivity of *als3* mutant, Gabrielson et al. (2006) identified a dozen of suppressor mutants, and two of them had different mutations on the same gene *ATR* (Rounds and Larsen, 2008). ATR (Ataxia Telangiectasia-mutated and Rad3-related) is a cell cycle checkpoint regulator that functions in detecting DNA damage and then halting cell division (Culligan et al., 2004). *atr* mutant is hypersensitive to clastogenic and genotoxic stresses, but shows increased tolerance to Al because of failure to halt cell cycle progression. Together with the recovery of the Al hypersensitivity of *als3* by the *atr* mutation, the results suggest that Al acts as a mild genotoxic agent and can target DNA to arrest root growth through ATR-regulated pathway (Rounds and Larsen, 2008).

In this study, to determine whether ATR-dependent pathway is required for the Al hypersensitivity in all Al-sensitive mutants, we created a series of double mutants between Al-sensitive mutants and *atr* mutant and then evaluated their sensitivity to Al in *Arabidopsis*. Our results revealed that the *atr* mutation could rescue the Al-sensitive phenotype of *als3*, *star1* and *als1*, but not that of *almt1* and *stop1*. These findings suggest that ATR-regulated pathway is required for internal Al toxicity-induced root growth inhibition.

## Materials and Methods

### Plant Materials and Growth Conditions

*Arabidopsis thaliana* (Columbia ecotype, Col-0) was used for all the control experiments. The T-DNA insertion lines *atr* (SALK_032841C), *star1* (GABI_762A06), *als3* (SALK_004094), *stop1* (SALK_114108), *almt1* (SALK_00962) and the mutant *als1-1* (CS3847) were all derived from uNASC^1^. Plants were grown in a growth chamber or controlled room at 22–25°C with 14 h of light and 10 h of darkness.

### Mutant Genotyping

To select homozygous mutants of *atr*, *als3*, *star1*, *stop1*, and *almt1*, primer pairs flanked each T-DNA insertion were used as follows: *ATR* (5′-ACTGCATGCCAT TTACTCCTAC-3′ and 5′-GATCAGCTTGATCATCCAAACT-3′), *ALS3* (5′- CAA TGTTCTTGCTCGTCCTCCT-3′ and 5′-TGGTTCACGTAGTGGGCCATCG-3′), *STAR1* (5′-TCGTAGAGTTGGAATGCTTTTTC-3′ and 5′-GTTGAAGAAACCTCTGTGCCATT-3′), *ALMT1* (5′-TTGAGAGAGCTGAGTGACCA-3′ and 5′-ACAAC GATATCAGCGCGAAC-3′), and *STOP1* (5′-TCTTAAAGCGGCCATTGGTG-3′ and 5′-TTAGAGACTAGTATCTGAAACAGACTCAC-3′). For *als1-1* mutant, a dCAPS (derive Cleaved Amplified Polymorphic sequences) marker was developed by using a primer pair (5′-TGTGAAACAGTTTGGTCGCT-3′ and 5′-TGCGTTTAGTCCTCCGAAGA-3′) and a restriction endonuclease TfiI. To generate double or triple mutants, crosses were made between *atr* and each Al-sensitive mutant or between *als3atr* and *star1* and then the derived F2 plants were genotyped and selected. For genotyping of *CyclinB1;1* and *QC46* marker lines, a primer pair for the *GUS* gene was used (5′-ATGTTACGTCCTGTAGAAACC-3′ and 5′-TCATTGTTTGCCTCCC TGCTGC-3′).

### RNA Isolation and Expression Analysis

Seeds were sterilized and stratified at 4°C for 2 days and then sowed on a 0.3% Gellan gum (G1910; Sigma–Aldrich) nutrient medium consisting of 1 mM KNO_3_, 0.2 mM KH_2_PO_4_, 2 mM MgSO_4_, 0.25 mM (NH_4_)_2_SO_4_, 1 mM Ca(NO_3_)2, 1 mM CaSO_4_, 1 mM K_2_SO_4_, 1 μM MnSO_4_, 5 μM H_3_BO_3_, 0.05 μM CuSO_4_, 0.2 μM ZnSO_4_, 0.02 μM NaMoO_4_, 0.1 μM CaCl_2_, 0.001 μM CoCl_2_ and 1% sucrose. After 7 days growth, the seedlings were transferred to a 0.5 mM CaCl_2_ solution for 6 h pretreatment at pH 4.8 and then exposed to a 0.5 mM CaCl_2_ solution (pH 4.8) with or without 20 μM AlCl_3_ for 12 h. Total RNA was extracted using TaKaRa MiniBEST plant RNA Extraction Kit (Cat # 9769). Around one microgram total RNA was first digested with DNase I and then subjected for the synthesis of first-strand cDNAs by using HiScript^®^ 1st Strand cDNA Synthesis Kit (Vazyme Biotech Co., Ltd., Nanjing, China). One twentieth of the cDNA products and the SYBR^®^ Green Master Mix kit (Vazyme Biotech Co., Ltd., Nanjing, China) were used for RT-PCR and real-time RT-PCR analysis. The primers for RT-PCR analysis of *ATR*, *ALS3*, *STAR1*, *ALMT1*, and *STOP1* were same to those primers for genotyping as shown above. The primers for real-time RT-PCR analysis were as follows: *ATR* (5′-CTGACTGAGGACTGTGGTCTGGT-3′ and 5′-GACGGTCACCAAGCCCAACA-3′), *ALS3* (5′-CGTATCTCTTCATGGTCTCTGTCG-3′ and 5′-GTAACTCCGGTGACGGTCATG-3′), *STAR1* (5′-TTCAAGGGACTGTTGCGGATA-3′ and 5′-AAGAGCACTTGTTGGTTCATCG-3′), *ALS1* (5′-GCCTCACAGTTGGTTCATCGG-3′ and 5′-GTCGTTTTTCCTCCACCGCT-3′), *ALMT1* (5′-TGCAAGCTGCGTTGTCGAC-3′ and 5′-CAAAATCTTGAAGGAAGTGGGAG-3′) and *STOP1* (5′-TCACATAGCTCTGTTCCAGGGA-3′ and 5′-ATCAGTCATTCCAGGCTGTGT-3′). *UBQ10* was used as an internal control and the forward and reverse prime sequences of *UBQ10* are 5′-CGTCTTCGTGGTGGTTTCTAA-3′ and 5′-GGATTATACAAGGCCCCAAAA-3′, respectively.

### Evaluation of Sensitivity to Al

For assessment of Al sensitivity in hydroponic conditions, we referred to a previous method with slight modifications (Huang et al., 2010). Briefly, seeds of each line were stratified at 4°C for 2 days and then sowed on a plastic mesh floating on a 1/30 strength Hoagland nutrient solution (NH_4_H_2_PO_4_ omitted) plus 1 mM CaCl_2_ and different concentrations of AlCl_3_ at pH 5.0 for 7 days. The solution was renewed every 3 days. After the treatment, the seedlings were photographed and root length was measured by ImageJ. Relative root growth expressed as (root length with Al treatment/root length without Al) × 100 was used to evaluate the Al sensitivity. For soaked gel experiments, we adopted the method developed by Larsen et al. (2005). Nutrient agar medium was first prepared, which consisted of 50 ml of 1 mM KNO_3_, 0.2 mM KH_2_PO_4_, 2 mM MgSO_4_, 0.25 mM (NH_4_)_2_SO_4_, 1 mM Ca(NO_3_)_2_, 1 mM CaSO_4_, 1 mM K_2_SO_4_, 1 μM MnSO_4_, 5 μM H_3_BO_3_, 0.05 μM CuSO_4_, 0.2 μM ZnSO_4_, 0.02 μM NaMoO_4_, 0.1 μM CaCl_2_, 0.001 μM CoCl_2_, 1% sucrose, and 0.3% Gellan gum (G1910; Sigma–Aldrich). The agar medium was then soaked with 25 ml of the same nutrient medium containing 0, 0.5, 0.75, or 1 mM AlCl_3_. After 2 days soaking, the solution was removed and seeds were grown on the agar medium plates for 7 days. The seedlings were then pictured and compared and the root length was measured by ImageJ.

### GUS Activity Assay

To investigate the effect of Al on Cyclin B1;1 accumulation, seeds of *CycB1;1:GUS* –containing WT, *atr*, *star1*, and *star1atr* were grown on a soaked gel medium containing 0 or 0.5 mM AlCl_3_ for 7 days. The seedlings were then stained with a commercialized GUS staining solution (161031; O’Biolab Co., Ltd., Beijing, China) for 2 h at 37°C. For determination of the status of the quiescent center (QC) after Al treatment, seeds of QC46 (GUS-based QC marker)-containing WT, *atr*, *als1*, and *als1atr* were grown on a soaked gel medium containing 0 or 1.5 mM AlCl_3_. After growth for 7 days, the seedlings were stained with the GUS staining solution overnight at 37°C. Stained tissues were observed and photographed with a microscope (Olympus BX53F, Japan).

## Results

### Mutation of *ATR* Rescued the Al-Sensitive Phenotype of Both *als3* and *star1* Mutants

**FIGURE 1:**
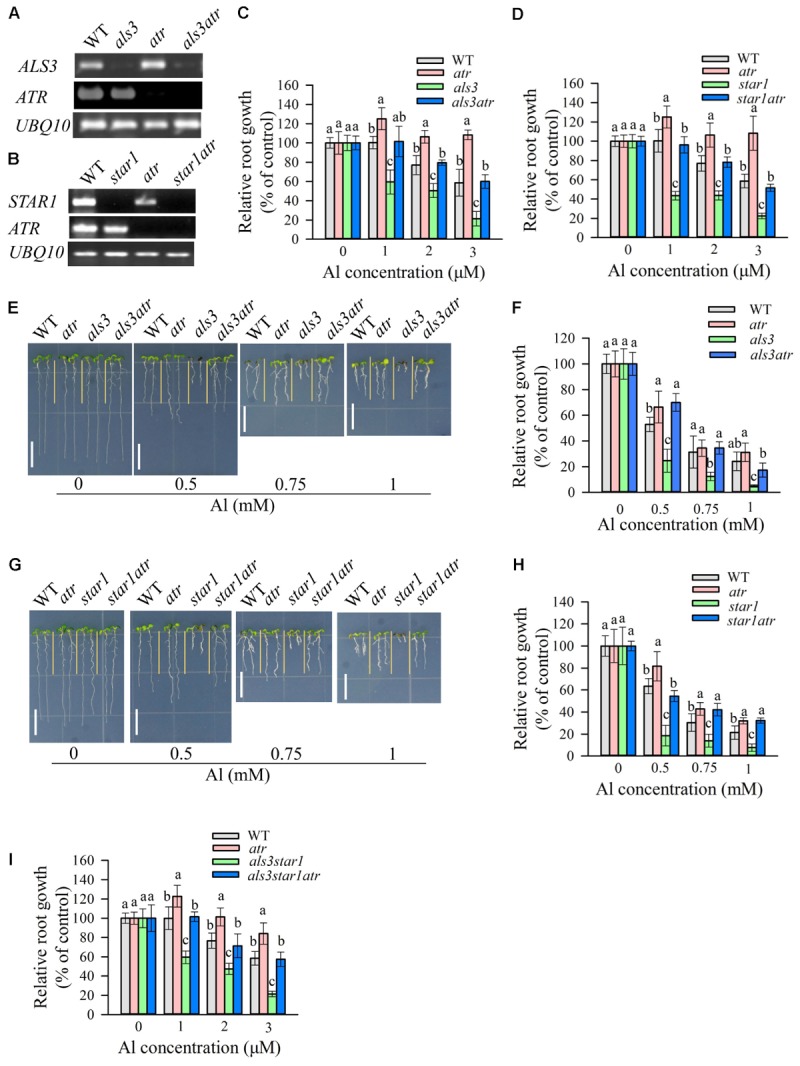
Rescue of the Al-sensitive phenotype of *als3* and *star1* by *atr* mutation. **(A,B)** RT-PCR analysis of *ATR*, *ALS3*, or *STAR1* in WT and different single or double mutants. UBQ10 was used as internal control. **(C,D)** Evaluation of Al tolerance in *als3* **(C)** or *star1* **(D)**-related mutants in hydroponic conditions. Seedlings were grown on a nutrient solution containing 0, 1, 2, or 3 μM Al at pH 5.0 for 7 days and then root length was measured and compared. Data are means ± SD (*n* = 15–20). **(E–H)** Evaluation of Al tolerance in soaked gel conditions. Seedlings were grown on a soaked gel medium containing 0, 0.5, 0.75, or 1 mM Al for 7 days. Data are means ± SD (*n* = 10–15). **(E,F)** Rescue of the Al-sensitive phenotype of *als3* by *atr*. **(G,H)** Rescue of the Al-sensitive phenotype of *star1* by *atr*. **(I)** Rescue of the Al-sensitive phenotype of *als3star1* by *atr* in hydroponic conditions. Means with different letters are significantly different (*P* < 0.05, Tukey’s test). Scale bar = 1 cm.

To confirm the previous observation that mutation of *ATR* could rescue the Al-sensitive phenotype of *als3* (Rounds and Larsen, 2008), we generated *als3atr* double mutant through a genetic cross between *atr* and *als3* single mutants. RT-PCR analysis revealed that *ATR* and *ALS3* were knocked out in respective single or double mutants (**Figure 1A**). We evaluated the tolerance of WT, *atr*, *als3*, and *als3atr* mutants to Al in both hydroponic and soaked gel conditions. Consistent with previous results, *atr* mutant showed more tolerance to Al than WT, and the *atr* mutation was able to reduce the sensitivity of *als3* to Al at all Al concentrations (**Figures 1C,E,F**). Nevertheless, mutation of *ATR* was not able to fully rescue the Al-sensitive phenotype of *als3*, especially at high Al concentrations (**Figures 1C,E,F**), suggesting that other Al toxicity mechanisms are also required for Al-induced growth inhibition in *als3* mutant. As STAR1 interacts with ALS3 to be involved in the regulation of Al tolerance in *Arabidopsis* (Huang et al., 2010; Dong et al., 2017), we investigated whether the *atr* mutation could also rescue the Al-sensitive phenotype of *star1*. We generated *star1atr* double mutant through crossing and genotyping and RT-PCR analysis confirmed that both *STAR1* and *ATR* were knocked out in the double mutant (**Figure 1B**). Evaluation of Al tolerance in the double mutant showed that *star1atr* was more tolerant to Al than *star1* at all Al concentrations (**Figures 1D,G,H**), indicating that ATR is required for Al-induced growth inhibition in *star1* mutant. Additionally, similar to that in *als3atr* mutant, mutation of *ATR* did not fully rescue the Al-sensitive phenotype of *star1* (**Figures 1D,G,H**). We also generated *star1als3* and *star1als3atr* mutants to further investigate whether mutation of *ATR* could rescue the Al sensitivity in *star1als3* double mutant. Results showed that the Al-sensitive phenotype of *star1als3* could also be rescued by the introduction of the *atr* mutation (**Figure 1I**). Together, these results confirm that STAR1 and ALS3 regulate Al tolerance through the same pathway and indicate that ATR-dependent pathway is also required for Al-induced growth inhibition in *star1* mutant.

**FIGURE 2:**
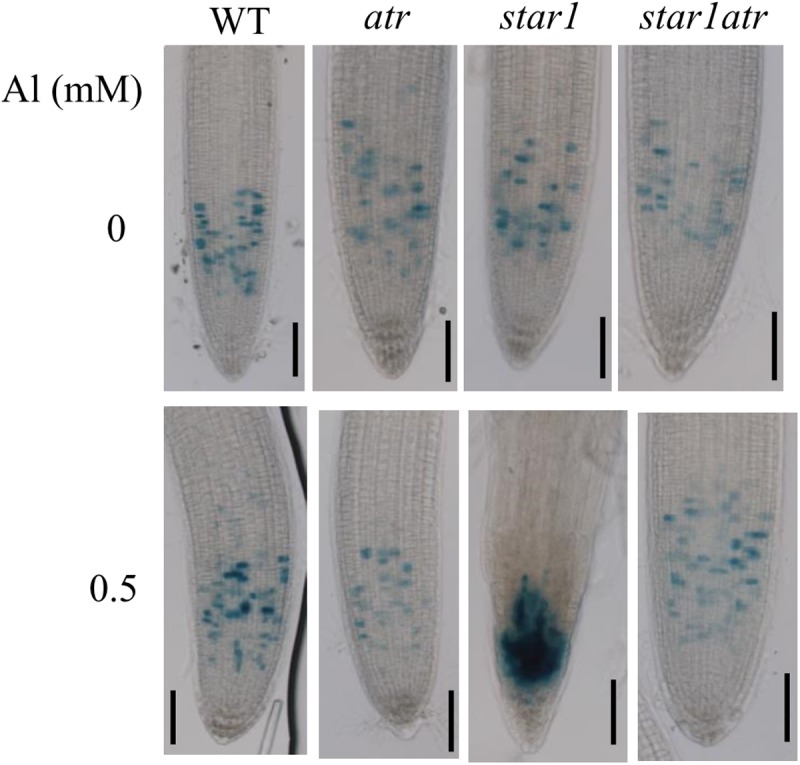
Rescue of cell cycle progression defects in *star1* by *atr* mutation under Al stress conditions. Seedlings of WT, *atr*, *star1*, and *star1atr* harboring *CycB1;1:GUS* marker were grown on a soaked gel medium containing 0 or 0.5 mM Al for 7 days and the roots were stained and observed under a microscope. Scale bar = 50 μm.

Al-induced inhibition of root growth was correlated with the increase in the number of cells trapped in the G2 stage, which causes the hyperaccumulation of Cyclin B1;1 in root tips (Rounds and Larsen, 2008). To examine the effect of Al on the accumulation of Cyclin B1;1 in *star1* mutant background, we introduced *CycB1;1:GUS* into *atr*, *star1* and *star1atr* through crossing. In the absence of Al, GUS expression was detected at relatively low levels in all the materials (**Figure 2**). After exposure to a low toxic level of Al, while GUS activity was slightly increased in WT, GUS expression in *star1* was dramatically increased in root tips, suggesting that cell cycle progression was halted in *star1* (**Figure 2**). In *star1atr*, GUS activity was detected at similar low levels to that in WT and *atr*, which suggested that the arrest of cell cycle progression in *star1* was rescued by the *atr* mutation. The Cyclin B1;1 expression results support the conclusion that knockout of *ATR* is able to rescue the Al hypersensitivity in *star1*.

### The *atr* Mutation Could Not Rescue the Al Hypersensitivity in Either *almt1* or *stop1* Mutants

**FIGURE 3:**
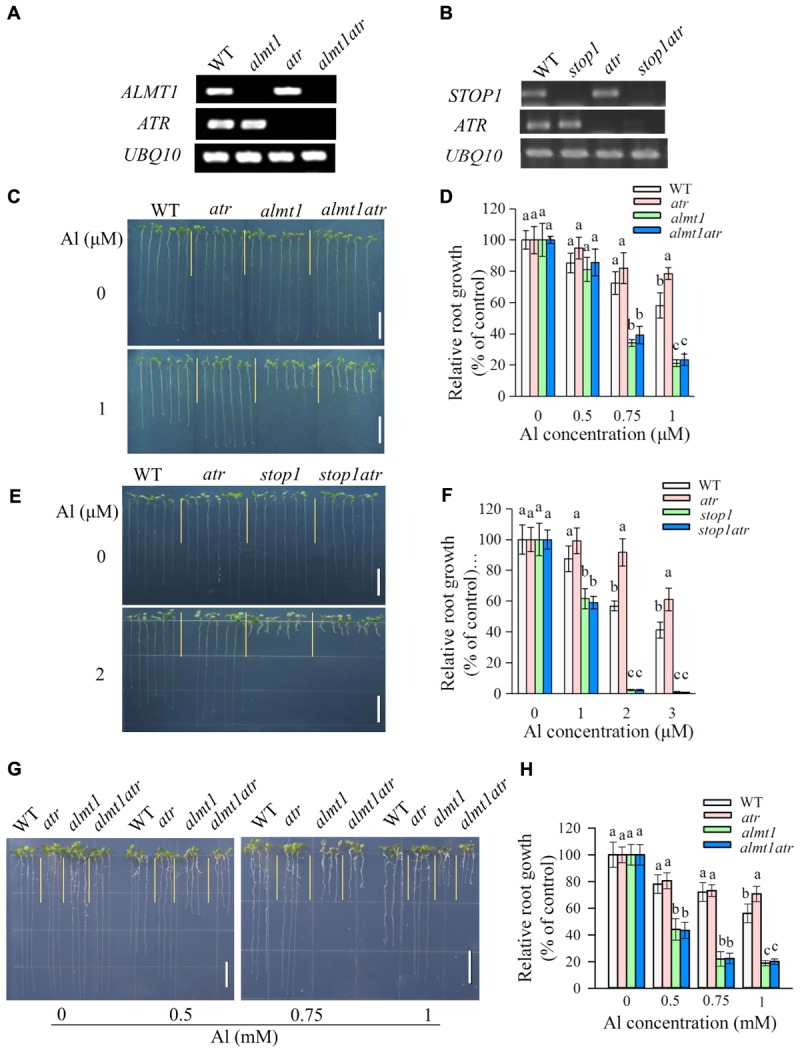
The Al hypersensitivity defects in *almt1* and *stop1* could not be rescued by the *atr* mutation. **(A,B)** RT-PCR analysis of *ATR*, *ALMT1*, or *STOP1* in WT and different single or double mutants. UBQ10 was used as internal control. **(C–F)** Evaluation of Al tolerance in *almt1* **(C,D)** or *stop1* **(E,F)**-related mutants in hydroponic conditions. Seedlings were grown on a nutrient solution with different concentrations of Al at pH 5.0 for 7 days and then root length was measured and compared. Data are means ± SD (*n* = 15–20). **(G,H)** Evaluation of Al tolerance in *almt1*-related mutants in soaked gel conditions. Seedlings were grown on a soaked gel medium containing 0, 0.5, 0.75, or 1 mM Al for 7 days. Data are means ± SD (*n* = 10–15). Means with different letters are significantly different (*P* < 0.05, Tukey’s test). Scale bar = 1 cm.

To investigate whether mutation of *ATR* could rescue the hypersensitivity of *almt1* and *stop1* to Al, we introduced the *atr* mutation into *stop1* and *almt1* mutants by crossing and genotyping, respectively. RT-PCR analysis confirmed that *ALMT1* or *STOP1* were knocked out in the corresponding mutants (**Figures 3A,B**). Phenotypic analysis of Al tolerance showed that the tolerance of *almt1atr* to Al did not differ from that of *almt1* at all Al concentrations in both hydroponic and soaked gel conditions (**Figures 3C,D,G,H**), indicating that mutation of *ATR* could not rescue Al-sensitive phenotype of *almt1*. Similarly, Al tolerance in *stop1atr* was also not different from that in *stop1* under all Al treatment (**Figures 3E,F**), demonstrating that the *atr* mutation was not able to rescue the Al-sensitive phenotype of *stop1* either. These results suggest that ATR is not required for Al-induced growth inhibition in those Al-sensitive mutants that are defective in the external detoxification of Al.

### The Al-Sensitive Phenotype of *als1* Could Also Be Rescued by the *atr* Mutation

**FIGURE 4:**
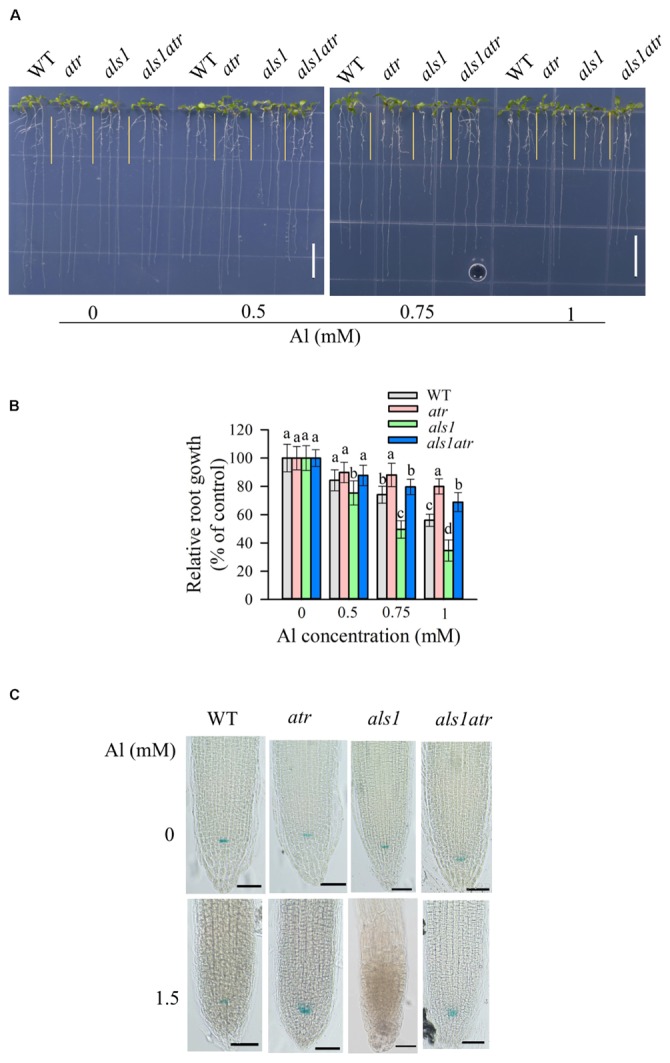
Rescue of the Al-sensitive phenotype of *als1* by *atr* mutation. **(A,B)** Seedlings of WT, *atr*, *als1*, and *als1atr* were grown on a soaked gel medium containing 0, 0.5, 0.75, or 1 mM Al for 7 days. Data are means ± SD (*n* = 10–15). Means with different letters are significantly different (*P* < 0.05, Tukey’s test). Scale bar = 1 cm. **(C)** Rescue of QC differentiation of *als1* by *atr* mutation. Seedlings of WT, *atr*, *als1*, and *als1atr* harboring *QC46* (QC-specific marker) were grown on a soaked gel medium containing 0 or 1.5 mM Al for 7 days and the roots were stained with GUS staining solution and observed under a microscope. Scale bar = 50 μm.

Since ATR is localized in the nucleus and required for Al-induced halting cell division in *als3* or *star1* (**Figures 1**, **2**), there are two possibilities that ATR might detect general internal Al toxicity signal or *star1*/*als3*-specific Al toxicity signal. To distinguish these two, we utilized another Al-sensitive mutant *als1*, which is deficient in the sequestration of Al into vacuoles (Larsen et al., 2007). Introduction of *atr* mutation into *als1* mutant could also rescue its Al-sensitive phenotype at various Al concentrations (**Figures 4A,B**). These results imply that ATR is required for internal Al toxicity-mediated root growth inhibition.

We also determined the status of the QC after Al treatment by introduction of a GUS-based QC marker, QC46 (Sabatini et al., 2003), into *atr*, *als1* and *als1atr*. Without Al treatment, GUS expression was well detected in all the materials (**Figure 4C**). However, in the presence of high levels of Al, GUS activity was lost in *als1*, suggesting that the essential stem cells required for maintenance of root growth was destroyed by Al toxicity in *als1* mutant. In contrast, *als1atr* double mutant displayed normal GUS activity in the QC after Al treatment (**Figure 4C**). These results indicate that the *atr* mutation could help *als1* mutant to maintain the QC integrity for root growth when exposure to highly toxic levels of Al.

### Expression Pattern of *ATR* and Al-Resistance Genes

**FIGURE 5:**
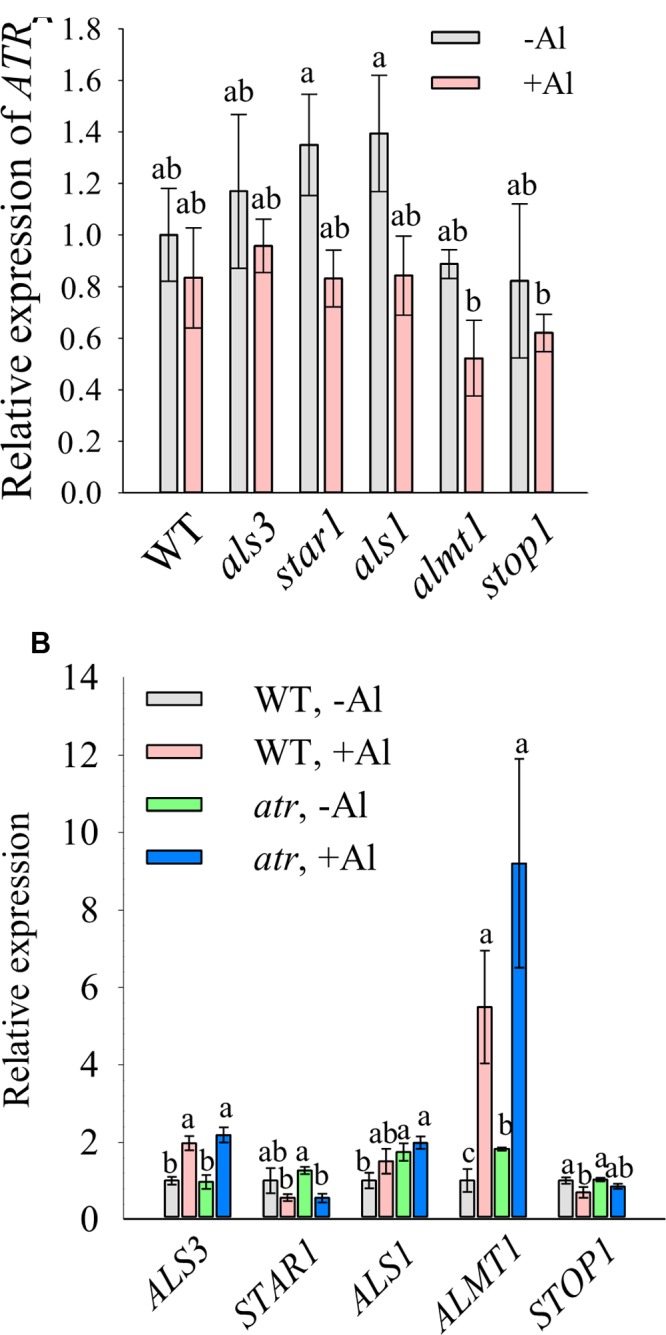
Expression analysis of *ATR* and Al-resistance genes. Seven-day-old seedlings grown on a nutrient agar medium were pretreated with a 0.5 mM CaCl_2_ solution for 6 h at pH 4.8 and then exposed to the same solution containing 0 or 20 μM Al at pH 4.8 for 12 h. The roots were sampled for expression analysis of *ATR* **(A)** or Al-resistance genes **(B)**. Data are means ± SD (*n* = 3). Means with different letters are significantly different (*P* < 0.05, Tukey’s test).

To examine whether *ATR* expression was altered in Al-sensitive mutants, we compared the expression level of *ATR* between WT and the Al-sensitive mutants. Results showed that there was no significant difference in *ATR* expression between WT and the mutants in the absence of Al (**Figure 5A**). Al treatment slightly decreased the expression of *ATR*, but no significant difference in *ATR* expression was found in WT and the mutants. This result suggests that increased Al sensitivity of the mutants was not due to altered *ATR* expression. The expression of Al-resistance genes in *atr* mutant was also determined. The expression levels of the Al-resistance genes including *ALS3*, *STAR1*, *ALS1*, *ALMT1*, and *STOP1* in *atr* mutant were similar to those in WT under both –Al and –Al conditions (**Figure 5B**), suggesting that increased Al tolerance in *atr* mutant was not caused by elevated expression of Al-resistance genes.

## Discussion

ATR functions as a cell cycle checkpoint to detect DNA damage and subsequently prevent cell division (Culligan et al., 2004). Since knockout of *ATR* is able to rescue the Al hypersensitivity in *als3* mutant (Gabrielson et al., 2006; Rounds and Larsen, 2008), two possible mechanisms exist for the increased sensitivity to Al in *als3*. One possible mechanism is that mutation of *ALS3* results in the increased Al accumulation in nucleus and consequently activates ATR-regulated pathway to halt cell division and ultimately inhibit root growth. The other is that Al toxicity-induced specific signal in *als3* activates ATR-regulated pathway to cause root growth inhibition. Our results showed that in addition to *als3*, mutation of *ATR* can also rescue Al-sensitive phenotype of *star1* and *als1*, indicating that rescue of Al-sensitive phenotype by *atr* mutation is not specific to *als3* mutant. Thus, we prefer the former hypothesis that elevated Al accumulation in the nucleus induces ATR-regulated pathway to inhibit root growth in *als3* mutant.

In contrast to its hypersensitivity to clastogenic and genotoxic stresses, *atr* mutant shows increased tolerance to Al. Al in nucleus might bind to DNA non-covalently and induce a conformational alteration from the B-form to Z-DNA, which affects DNA unwinding during DNA replication (Anitha and Rao, 2002). Nevertheless, unlike other genotoxic stresses, Al is thought to be a mild DNA damage agent and its binding to DNA is likely to be reversible (Rounds and Larsen, 2008; Nezames et al., 2012). This unique interaction of Al with DNA can activate ATR-, ALT2-, and SOG1-regualted transcriptional response to halt cell division and cause the inhibition of root growth (Sjogren et al., 2015). However, it remains unknown about how the interaction of Al with DNA activates the ATR-regulated pathway and what the ATR-regulated downstream transcriptional events that lead to the cease of cell division are.

The inhibition of root growth can be attributed to the disruption of cell division and/or cell elongation. Rapid reduction in root growth suggests an initial impact of Al on cell elongation instead of cell division (Sharp et al., 1988; Kopittke et al., 2015). However, when roots are exposed to Al for a long period of time, inhibition of cell division might also contribute to the reduction of root growth. Al-activated ATR-regulated cease of cell division in *als3*/*star1* or *als1* suggests that inhibition of cell division plays a critical role in Al-induced inhibition of root growth in these Al-sensitive mutants. Further work is required to determine whether mutation of *atr* could rescue the Al-sensitive phenotype of these mutants after a short-term exposure to Al.

Numerous studies have suggested that Al can target multiple sites for toxicity, including apoplastic and symplastic components (Kochian, 1995; Ma, 2007). Nevertheless, it remains debatable about which sites play more important roles in Al-induced inhibition of root growth. We found that the *atr* mutation could not rescue the Al hypersensitivity in *almt1* and *stop1*, which are defective in the capacity to detoxify Al externally. These results indicate that ATR is not required for Al-induced inhibition of root growth in all Al-sensitive mutants and suggest that both symplastic components such as DNA and apoplastic components including cell wall are important Al target sites that lead to root growth inhibition by Al toxicity. Additionally, our data showed that the *atr* mutation could not fully rescue the Al hypersensitivity in *als3*, suggesting that Al also targets other symplastic sites to cause root growth inhibition in *als3* mutant.

In rice, OsSTAR1 interacts with OsSTAR2, the rice ortholog of ALS3, to form a functional complex that is suggested to be involved in the modification of cell wall that is required for Al detoxification (Huang et al., 2009). Although *Arabidopsis* AtSTAR1 can also interact with ALS3 to be involved in the detoxification of Al, AtSTAR1 and ALS3 are localized to tonoplast (Larsen et al., 2005; Huang et al., 2010; Dong et al., 2017), which are different from OsSTAR1 and OsSTAR2 that are localized to vesicle membranes (Huang et al., 2009). We found that in addition to *als3*, knockout of *ATR* also rescues the Al-sensitive phenotype of *star1*. Furthermore, the *atr* mutation can even rescue Al hypersensitivity in *als3star1* double mutant. These results indicate that *als3* and *star1* share the same mechanism for their hypersensitivity to Al, i.e., ATR-regulated pathway required for Al-induced inhibition of root growth. The results also support the view that STAR1 and STAR2/ALS3 function together to be involved in the same pathway of Al detoxification. We further found that the Al hypersensitivity in *als1* was rescued by the *atr* mutation. *als1* has defects in the internal detoxification of Al (Larsen et al., 2007). Together, our results suggest that ATR is required for internal Al toxicity-induced inhibition of root growth and that STAR1 and ALS3 might be involved in the internal detoxification of Al in *Arabidopsis*. We propose that under Al stress conditions, internal Al detoxification-deficient mutants accumulate high levels of Al in the nucleus, which induces DNA damage and consequently activates ATR-regulated pathway and arrest cell cycle, finally leading to the inhibition of root growth.

## Author Contributions

All authors conceived the project. C-FH drafted the manuscript. YZ, JG, MC, LL, and LW performed the experiments. YZ and JG helped to analyze the data and write the manuscript. All authors read and approved the final manuscript.

## Conflict of Interest Statement

The authors declare that the research was conducted in the absence of any commercial or financial relationships that could be construed as a potential conflict of interest.

## Footnotes

1 http://szlapncs01.nottingham.ac.uk/
