# Corrigendum: The Cell Cycle Checkpoint Regulator ATR Is Required for Internal Aluminum Toxicity-Mediated Root Growth Inhibition in *Arabidopsis*

**DOI:** 10.3389/fpls.2018.00316

**Published:** 2018-03-09

**Authors:** Yang Zhang, Jinliang Guo, Mo Chen, Lun Li, Lihua Wang, Chao-Feng Huang

**Affiliations:** ^1^College of Resources and Environmental Sciences, Nanjing Agricultural University, Nanjing, China; ^2^Shanghai Center for Plant Stress Biology, National Key Laboratory of Plant Molecular Genetics, CAS Center for Excellence in Molecular Plant Sciences, Chinese Academy of Sciences, Shanghai, China; ^3^Flower Research Institute, Yunnan Academy of Agricultural Sciences, Kunming, China

**Keywords:** aluminum toxicity, *Arabidopsis thaliana*, ATR, cell cycle checkpoint, DNA damage, external, internal

There were mistakes in the icon colors (right upper corner) of Figures 1C,D,H,I, 3D,F,H, 4B. The correct version of these figures appears below. The authors apologize for the mistakes. This error does not change the scientific conclusions of the article in any way.

**Figure 1:**
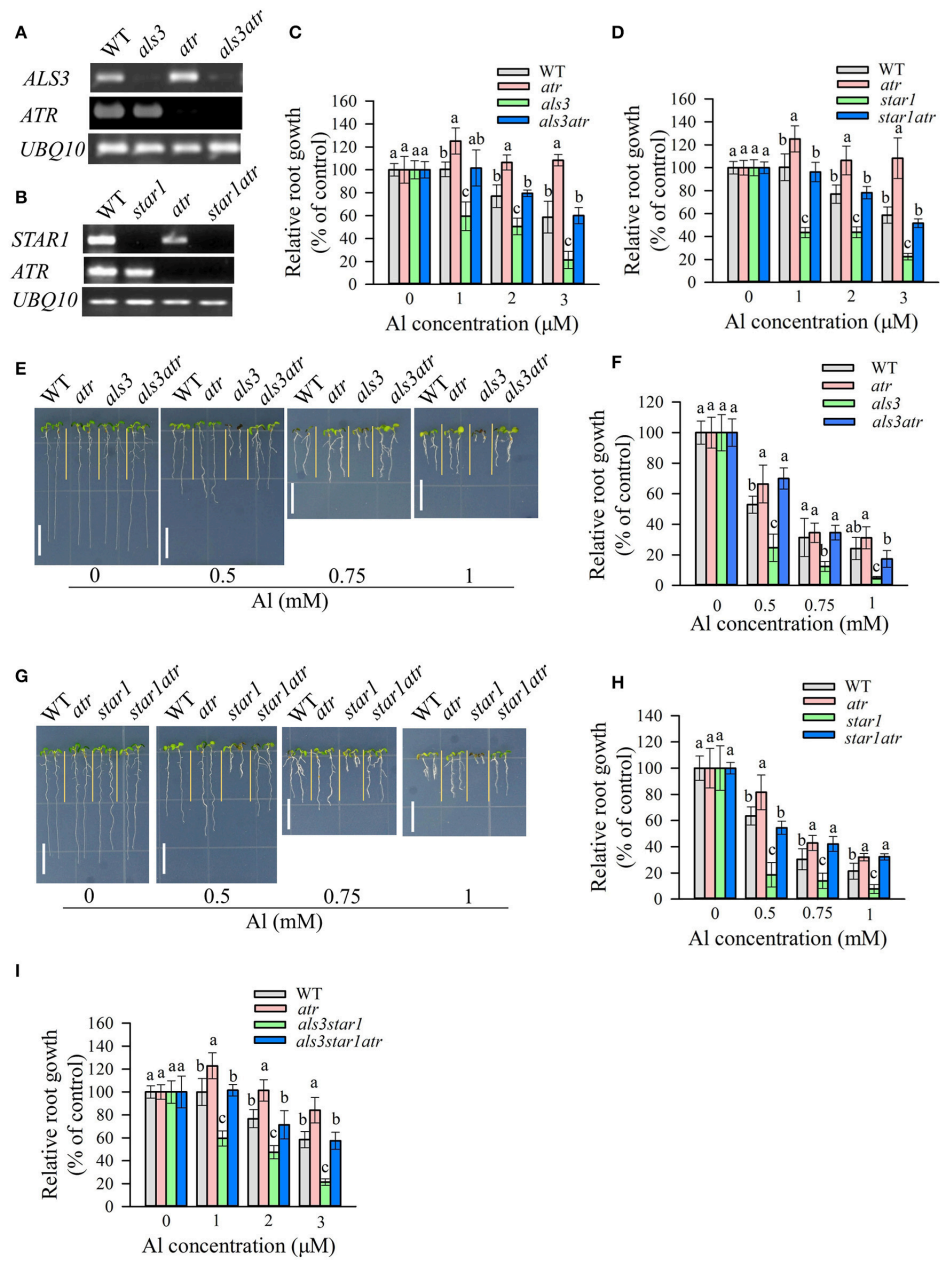
Rescue of the Al-sensitive phenotype of *als3* and *star1* by *atr* mutation. **(A,B)** RT-PCR analysis of *ATR, ALS3*, or *STAR1* in WT and different single or double mutants. UBQ10 was used as internal control. **(C,D)** Evaluation of Al tolerance in *als3* **(C)** or *star1* **(D)**-related mutants in hydroponic conditions. Seedlings were grown on a nutrient solution containing 0, 1, 2, or 3 μM Al at pH 5.0 for 7 d and then root length was measured and compared. Data are means ± SD (*n* = 15–20). **(E–H)** Evaluation of Al tolerance in soaked gel conditions. Seedlings were grown on a soaked gel medium containing 0, 0.5, 0.75, or 1 mM Al for 7 d. Data are means ± SD (*n* = 10–15). **(E,F)** Rescue of the Al-sensitive phenotype of *als3* by *atr*. **(G,H)** Rescue of the Al-sensitive phenotype of *star1* by *atr*. **(I)** Rescue of the Al-sensitive phenotype of *als3star1* by *atr* in hydroponic conditions. Means with different letters are significantly different (*P* < 0.05, Tukey's test). Scale bar = 1 cm.

**Figure 3:**
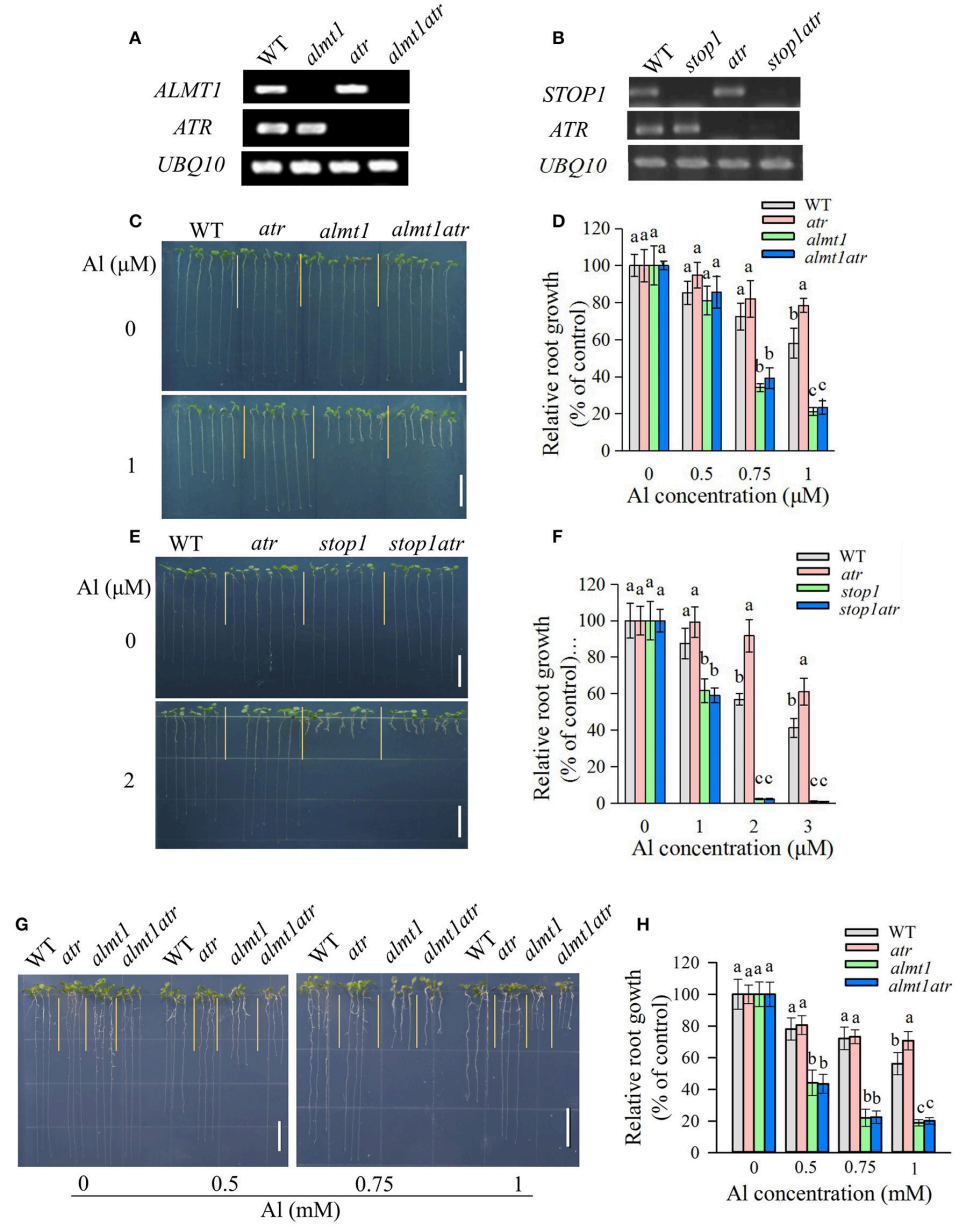
The Al hypersensitivity defects in *almt1* and *stop1* could not be rescued by the *atr* mutation. **(A,B)** RT-PCR analysis of *ATR, ALMT1*, or *STOP1* in WT and different single or double mutants. UBQ10 was used as internal control. **(C–F)** Evaluation of Al tolerance in *almt1* **(C,D)** or *stop1* **(E,F)**-related mutants in hydroponic conditions. Seedlings were grown on a nutrient solution with different concentrations of Al at pH 5.0 for 7 d and then root length was measured and compared. Data are means ± SD (*n* = 15–20). **(G,H)** Evaluation of Al tolerance in *almt1*-related mutants in soaked gel conditions. Seedlings were grown on a soaked gel medium containing 0, 0.5, 0.75, or 1 mM Al for 7 d. Data are means ± SD (*n* = 10–15). Means with different letters are significantly different (*P* < 0.05, Tukey's test). Scale bar = 1 cm.

**Figure 4:**
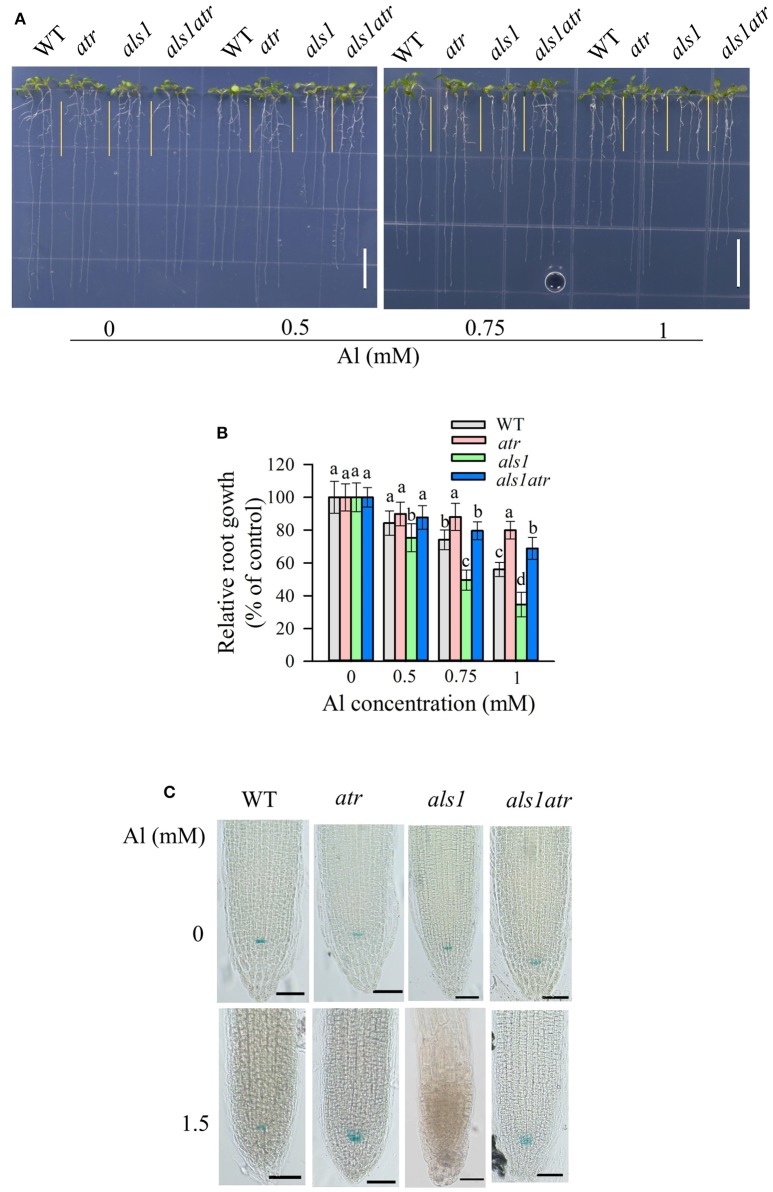
Rescue of the Al-sensitive phenotype of *als1* by *atr* mutation. **(A,B)** Seedlings of WT, *atr, als1*, and *als1atr* were grown on a soaked gel medium containing 0, 0.5, 0.75, or 1 mM Al for 7 d. Data are means ± SD (*n* = 10–15). Means with different letters are significantly different (*P* < 0.05, Tukey's test). Scale bar = 1 cm. **(C)** Rescue of QC differentiation of *als1* by *atr* mutation. Seedlings of WT, *atr, als1*, and *als1atr* harboring *QC46* (QC-specific marker) were grown on a soaked gel medium containing 0 or 1.5 mM Al for 7 d and the roots were stained with GUS staining solution and observed under a microscope. Scale bar = 50 μm.

The original article has been updated.

## Conflict of interest statement

The authors declare that the research was conducted in the absence of any commercial or financial relationships that could be construed as a potential conflict of interest.

